# Local ecological knowledge and its relationship with biodiversity conservation among two *Quilombola* groups living in the Atlantic Rainforest, Brazil

**DOI:** 10.1371/journal.pone.0187599

**Published:** 2017-11-28

**Authors:** Bruno Esteves Conde, Tamara Ticktin, Amanda Surerus Fonseca, Arthur Ladeira Macedo, Timothy Ongaro Orsi, Luciana Moreira Chedier, Eliana Rodrigues, Daniel Sales Pimenta

**Affiliations:** 1 Departamento de Botânica, Universidade Federal de Juiz de Fora, Juiz de Fora, Minas Gerais, Brazil; 2 Botany Department, University of Hawai’i at Mānoa, Honolulu, Hawaii, United States of America; 3 Departamento de Ciências Biológicas, Centro de Ensino Superior de Juiz de Fora, Juiz de Fora, Minas Gerais, Brazil; 4 Departamento de Química Orgânica, Universidade Federal Fluminense, Niterói, Rio de Janeiro, Brazil; 5 Departamento de Geografia, Universidade Federal de Juiz de Fora, Juiz de Fora, Minas Gerais, Brazil; 6 Departamento de Ciências Ambientais, Universidade Federal de São Paulo, Diadema, São Paulo, Brazil; Oklahoma State University, UNITED STATES

## Abstract

Information on the knowledge, uses, and abundance of natural resources in local communities can provide insight on conservation status and conservation strategies in these locations. The aim of this research was to evaluate the uses, knowledge and conservation status of plants in two Quilombolas (descendants of slaves of African origin) communities in the Atlantic rainforest of Brazil, São Sebastião da Boa Vista (SSBV) and São Bento (SB). We used a combination of ethnobotanical and ecological survey methods to ask: 1) What ethnobotanical knowledge do the communities hold? 2) What native species are most valuable to them? 3) What is the conservation status of the native species used? Thirteen local experts described the names and uses of 212 species in SSBV (105 native species) and 221 in SB (96 native species). Shannon Wiener diversity and Pielou’s Equitability indices of ethnobotanical knowledge of species were very high (5.27/0.96 and 5.28/0.96, respectively). Species with the highest cultural significance and use-value indexes in SSBV were *Dalbergia hortensis* (26/2.14), *Eremanthus erythropappus* (6.88/1), and *Tibouchina granulosa* (6.02/1); while *Piptadenia gonoacantha* (3.32/1), *Sparattosperma leucanthum* (3.32/1) and *Cecropia glaziovii* (3.32/0.67) were the highest in SB. Thirty-three native species ranked in the highest conservation priority category at SSBV and 31 at SB. *D*. *hortensis* was noteworthy because of its extremely high cultural importance at SSBV, and its categorization as a conservation priority in both communities. This information can be used towards generating sustainable use and conservation plans that are appropriate for the local communities.

## Introduction

Brazil is one of the world’s megadiverse countries, and the Atlantic rainforest, which stretches from the northeastern to the southern regions of the country, is the most biodiverse biome of Brazil, with up to 476 plant species found in one hectare [1]. Unfortunately, the Atlantic rainforest is also one of the most threatened forest types in the world, with nearly 90% of its original area devastated [2]. As is the case with the majority of Brazilian protected areas [3], the Atlantic Rainforest is also home to many traditional communities–those that have lived in one location for a long period of time, such as the *Quilombolas*. According to the Living Report of World Wide Fund for Nature [4], 90% of tropical forests worldwide are not under formal protection and millions of people living both inside and outside of reserves rely on their resources [5].

The *Quilombolas* are descendants of slaves of African origin who came to Brazil during the colonial (1530–1815), united kingdom (1815–1822) and empire (1822–1889) periods. Some of these slaves fled the farms where they were exploited, organizing communities of refugees, called *Quilombolas*, in the local forests. Since that time, the *Quilombolas* have lived in villages where they have made a living from agriculture and use of forest resources. Like other traditional communities, over time they have developed detailed local ecological knowledge systems (LEK) [6, 7]. LEK systems are knowledge practice and belief systems about the relationships of living beings, including humans, with one another and with their environments. LEK is developed through the process of observation and experimentation and is passed down through generations [8, 9]. Research outside of Brazil has shown that communities of freed or escaped slaves, also known as maroons, have high levels of knowledge of plants [10], and strong conservation practices for their natural resources [11].

It is important for communities, such as the *Quilombolas*, who continue to depend on the local environment as a primary source of resources, to develop the means to maintain and preserve local species. Understanding LEK, including ethnobotanical knowledge and natural resource use strategies, is critical to developing strategies for conservation [12]. Conservation projects that do not include communication with and/or participation of local communities who use the resources can be problematic. In addition, the loss of local knowledge and practices may compromise not only cultural knowledge but also local biodiversity [13]. Surveys of useful plant resources can provide information to help evaluate conservation status and the potential for sustainable use [14]. In Brazil, little is known about the knowledge, use, and conservation of resources of *Quilombolas* communities. Crepaldi and Peixoto [15] documented species abundance in forests and how they were managed in a *Quilombola* community in the state of Espírito Santo, Brazil, but beyond this study little information is available. Similarly, França [16], documented the species in Campinho da Independência, Paraty/RJ, and Avila, Zank [17] the species of three communities in Santa Catarina.

This work focused on two *Quilombolas* communities in the Atlantic forest of Minas Gerais state in Brazil to address the following questions: 1) What ethnobotanical knowledge do the communities hold? 2) What are native plant species most valuable to them? 3) What is the conservation status of the native species used? By developing a list of local forest species and their conservation status, we also aimed to identify species at risk [18], and therefore generate some of the information needed for sustainable management plans.

## Methods

### Study sites

**Fig 1:**
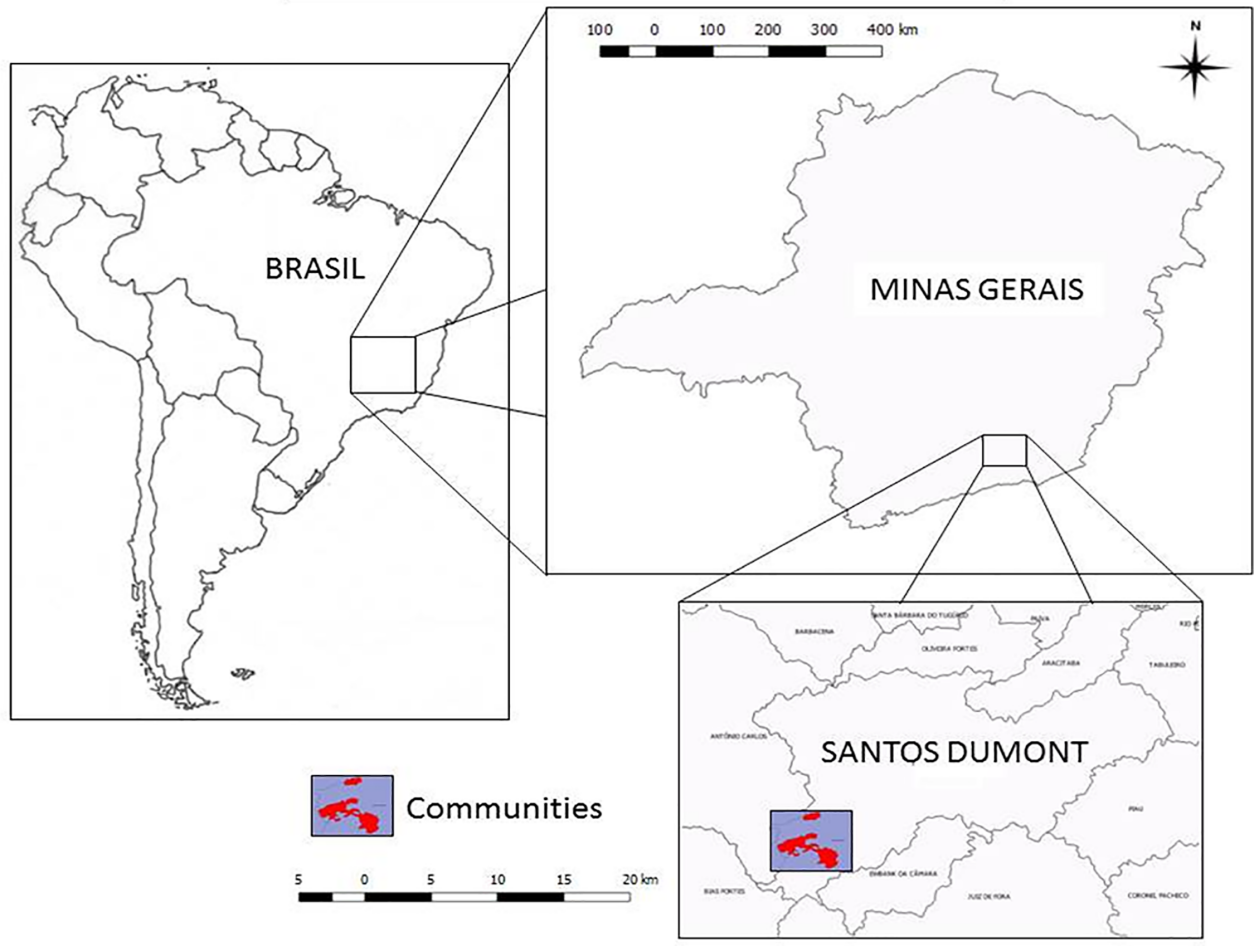
Localization of the communities studied, São Sebastião da Boa Vista (SSBV) and São Bento (SB). Santos Dumont city, Minas Gerais state/Brazil.

We carried out our research in two *Quilombolas* communities located inside the Atlantic Rainforest in Minas Gerais state of Brazil: São Sebastião da Boa Vista (SSBV) (21°31’0.24” S e 43° 39’ 30. 26” W) and São Bento (SB) (21° 33’ 39.33 S e 43° 38’ 59. 94” W) (Fig 1). The vegetation in these communities range from grassland to forest to *Eucalyptus* plantations, as well as farms with crops and cattle. Historically, these farms were run by slave owners, and the *Quilombolas* are descendants of those slaves. Today, most of the inhabitants continue to raise crops and cattle on their land, but some young people work as wage laborer in eucalyptus farms in the surrounding areas.

Since 2010 both communities have had linkages with the Geosciences department/Geography and Botany department/ICB at the Federal University of Juiz de Fora. The communities of SSBV and SB provide excellent locations to study local ecological knowledge as they have been partially isolated for many years, exclusively using the natural resources around them, and so and have developed much knowledge about the use of the forest surrounding the communities.

**Fig 2:**
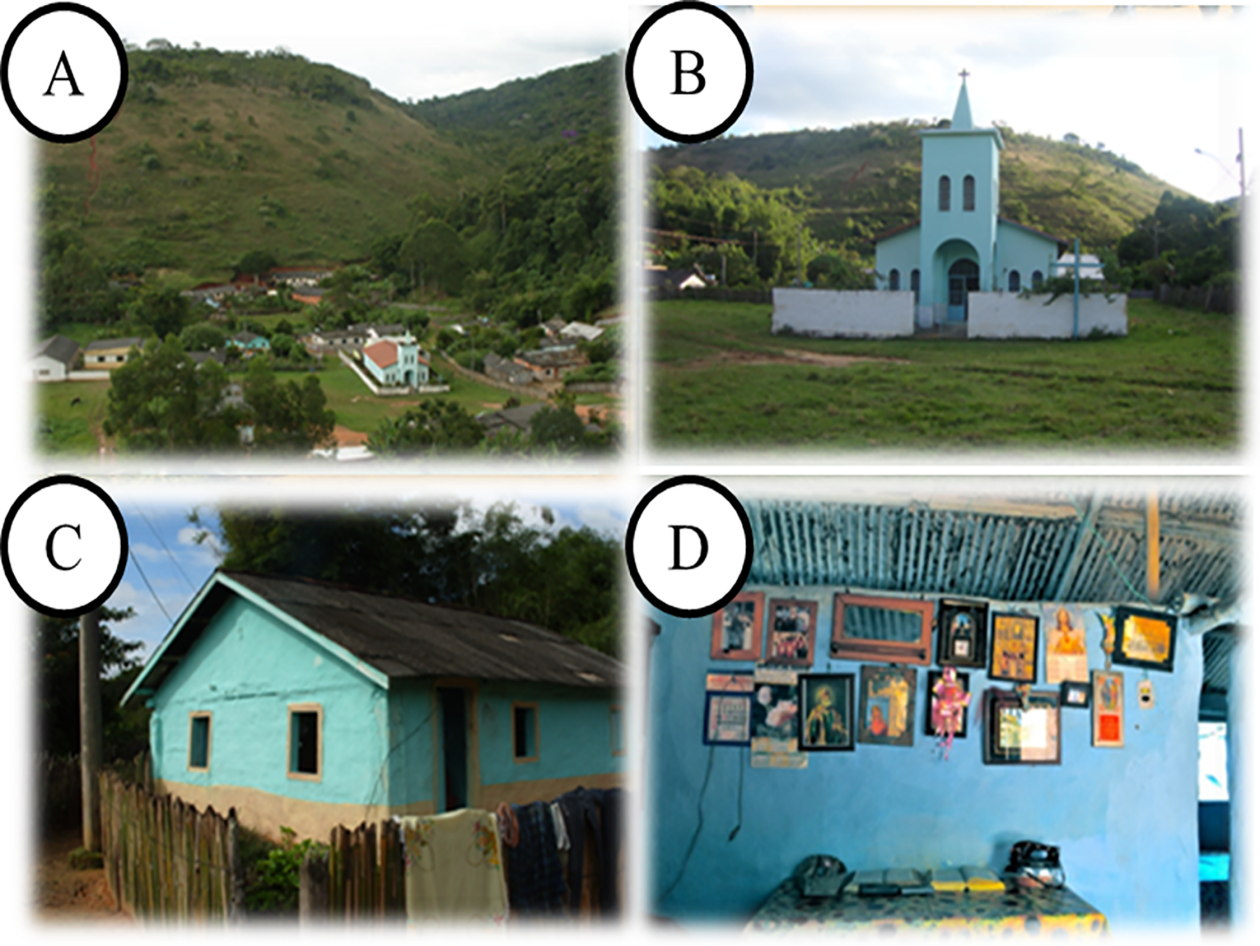
São Sebastião da Boa Vista community. A: View about a community; B: Church of São Sebastião da Boa Vista; C and D: Common style house in the community.

**Fig 3:**
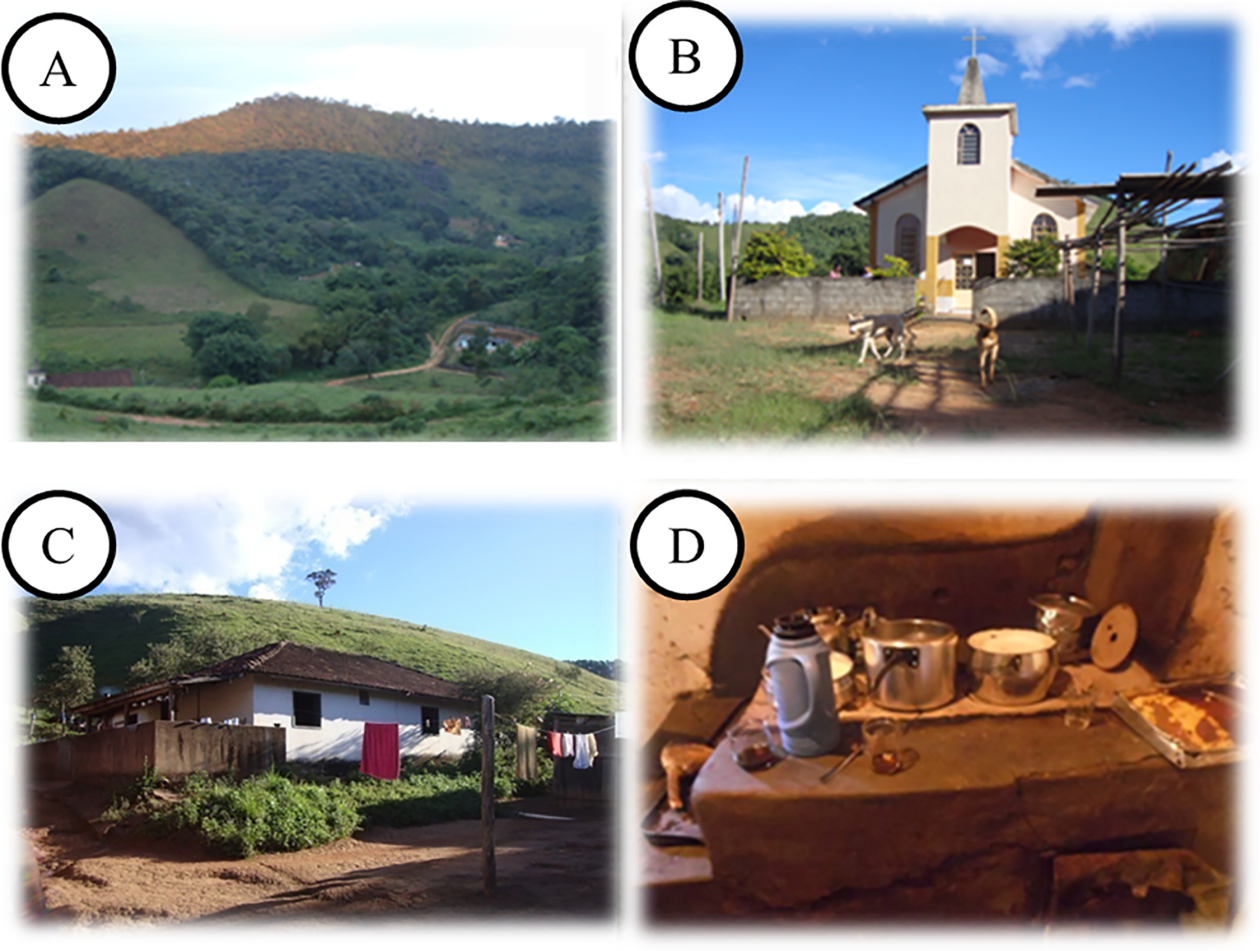
São Bento community. A: View of the community; B: Church of São Bento; C and D: Common style house in the community.

The size of the communities’ territories are: 130 hectares (SSBV) and 8000 hectares (SB). At SSBV, houses are located at the community center, surrounding the church in a radius of at most 300 m. Today the community has 36 houses and 98 inhabitants. At SB, houses are further away from each other, but the church is considered the community center and the meeting point of villagers. Presently, the community has 20 houses and 85 inhabitants; houses are scattered around the woods in a radius of up to 6 km and they have restricted access by trails (Figs 2 and 3).

Catholic churches are the main places of worship for the communities; however, elements of African religions are present, demonstrating religious syncretism.

### Ethnography, consent and ethical approval

We made ten trips were made to each community between March and December of 2012. These trips included home visits to all houses in each community for informal interviews with the inhabitants and participant observation [19]—observing and participating in daily activities with the residents.

Home visits were carried out together with a key informant, who contributed actively to the research [20]. The main discussions were about life histories, local daily problems, collective life, and health. We also which community members were experts in health and/or knowledge of plants [21].

At the end of this stage, participants signed the free, prior and informed consent agreement provided by the Brazilian Ministry of Culture.

Permission to conduct this study was obtained from “Instituto do Patrimônio Histórico e Artístico Nacional” (IPHAN–Nacional Institute of Historic and Artistic Patrimony) by permit n°01450.010839/2012-62 (S1 Appendix). To obtain this permission, a meeting with all the community members, recorded in the minutes of the residents’ association, were made at each *Quilombola*, when all steps of the work were explained, prevising the participation of citizens of all age groups. In these meetings the president of the residents’ association signed a Consent Form provided by IPHAN on behalf of the whole community, authorizing the research at the *Quilombolas* and with their citizens. After that, these Consent Forms were sent to IPHAN and the permission was obtained.

### Collection of ethnobotanical data

Ethnobotanical data were collected through interviews with local experts, where the snow ball method [19] was employed, and local experts indicated other possible plant experts. A total of 13 local experts were identified. The group in SSBV was of 7 experts (2 men and 5 women) and in SB was of 6 experts (2 men and 4 women). The age of these specialists ranged from 26 to 84 years, and their social occupations included traditional cooks, builders, craftsmen, spiritual healers, lumberjack and/or bushman (Table 1).

**Table 1:** Gender, age, and number of local specialists with knowledge of different plant use categories in São Sebastião da Boa Vista (SSBV) and São Bento (SB).

Community	Gender		Specialty categories							Average age ± SD
	M	F	MP	TC	Bu	Cr	SH	Lu	Bm	
São Sebastião da Boa Vista	2	5	7	2	2	1	2	2	2	58.7 ± 9.7
São Bento	3	3	5	2	2	1	2	2	2	67.1 ± 3.9
Total	5	8	12	4	4	2	4	4	4	-
Average of the averages	-	-	-	-	-	-	-	-	-	62.9

(M) = Male; (F) = Female; (MP) = Knowledge of medicinal plants; (TC) = Traditional cooks; (Bu) = Builders; (Cr) = Craftsman; (SH) = spiritual healers, that have supernatural power to cures and other spells; (Lu) = Lumberjack; (Bm) = Bushman = main collectors of raw forest material.

Interviews using semi-structured questionnaires were carried out with local experts [22] where they were asked about the use of plants for all purposes (Table 2).

**Table 2:** Plant uses by *Quilombolas* of São Sebastião da Boa Vista (SSBV) and São Bento (SB)–listing by categories adapted from Galeano [23].

Use category	Use type
Food	Heart of palm
	Leaves, fruits, and flowers eaten raw or cooked
	Fruits used for production of alcoholic beverages
	Edible fruits
	Spices
Building	House found
	Flooring
	Pillars
	Crafting
	Thatched roof
Fuel	Fire production (for multiple purposes)
Medicinal	Medicines
Ornamental	Grown for ornamentation
Ritualistic	Bath to discharge the body of bad energy
	Protect the house
Technology	Sarong making
	Fishing tools
	Furniture
	Cable tools in general
	Stakes and fences
	Handicrafts for decoration
	Kitchenware

To triangulate the information collected in interviews, focus group discussions were carried out with the whole community in day-long meetings (1 in each community). We directly invited all households to attend (by going door to door). The focus group in SSBV was made up of 18 teenagers (12–18 years old; ten female and eight male), 16 adults (over 18 and less than 60 years old; nine women and seven men) and nine elders (over 60 years old; five women and four men). In SB there were 20 teenagers (15 female and five male), ten adults (seven women and three men) and eight elders (six women and two men). The ages ranged from 18 to 66 in SSBV and 18 to 75 in SB. Focus group discussions focused on the vernacular names of plants and their use categories (Table 2). Pictures or *in vivo* specimens were presented and participants openly discussed the plants used. All participants present had the opportunity to participate. Focus groups lasted up to one hour.

### Collection and identification of plant specimens

After obtaining ethnobotanical data, fertile species were collected *in vivo* [24] by the “walk in the woods method” [25] with local experts. Voucher specimens were prepared and identified by experts from Universidade Federal de Juiz de Fora (UFJF) and partner specialists and vouchers were deposited in Leopoldo Krieger Herbarium (CESJ). Scientific names and families of species were checked using theplantlist.org

In cases where the flowering period did not coincide with the field visits, non-fertile species were collected but were identified by comparison with samples of CESJ Herbarium and with image records of Virtual Herbarium of Musém National d’Historie Naturelle, Royal Botanical Gardens, and Missouri Botanical Garden. For those plant species for which it was not possible to collect samples, the checklist method was performed [22]. Botanical species photographs from the Ethnobotanical Laboratory of UFJF collection were shown to interviewees so that they could confirm which ones they had cited in the surveys and focus groups.

### Evaluation of origin and conservation status of plants used in the communities

Information about the species named and collected was searched for in the Flora Brasiliensis [26], The Botanical List of Brazilian Species (Reflora) and the Native Species Manual [27]. For evaluation of conservation status, only native species were considered. For Atlantic rainforest species that are harvested, information on the conservation status and threats were searched for using the following databases: Ministério do Meio Ambiente, Biodiversitas Foundation and International Union for Conservation Nature.

### Data analyses

To evaluate ethnobotanical knowledge homogeneity and diversity of the study communities, Pielou’s Equitability index (EI) and Shannon-Wiener’s biological diversity index (BDI) were used [28]. These indices, commonly used in ecology, have been adapted to ethnobotany to evaluate the uniformity and diversity of ethnobotanical knowledge respectively, of a particular community. These indices were calculated based on every species of the ethnobotanical collection in both communities; native and exotic species were both included. The software PAST v.134 [29] and the equations below were used:

Shannon-Wiener Index

H'=−∑Pi×logPi

Where:

*Pi* = *n*^*i*^/*N*
*H*' = *BDI*
n^i^ = only citations per species only from the interviews
N = total of citations

Pielou’s Equitability index:

$$J'=\frac{H'}{H{'}_{\max}}$$

*BDI* = *H*'
H’_max_ = (natural base logarithm) of total species number

These indices were also compared with those found from other studies in Brazil.

To measure the importance of each native species, we used the Cultural Significance index (CSI) [30]:

CSI=∑(i×e×c)×CF

i = species management (ranging between 1 and 2. Being 2 = cultivated or managed)
e = preferential use (ranging between 1 and 2. Being 2 = preferential for a particular use)
c = use frequency (ranging between 1 and 2. Being 1 for rarely cited—cited by less than two people or under 10% of citation)
CF = correction factor (citations of species x/citations of the most cited species)
* (i x e x c) = must be calculated for each use category

**Fig 4:**
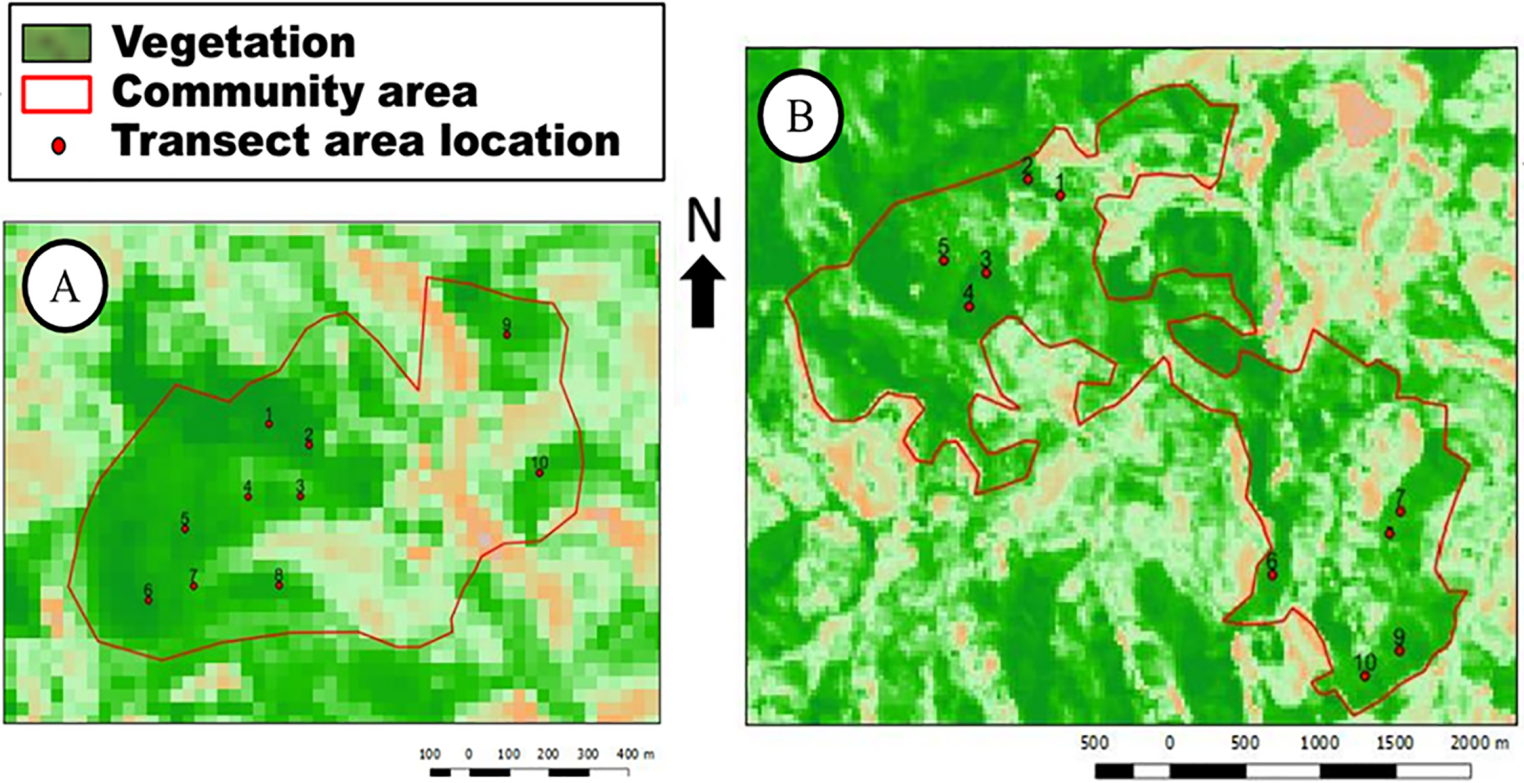
Aerial overview of the communities. A: São Sebastião da Boa Vista; B: São Bento.

To assess the conservation status of native forest plant species used by SSBV and SB communities, we adapted the Conservation Priority Index (CPI) [18], which considers the following criteria: sampled density, risk based on collection type, local importance and diversity of uses. The forests area for each community was large (40.000 m^2^ in SSBV and 150.000 m^2^ in SB), therefore plots were established to obtain species densities. As suggested by Espírito-Santo, Shimabukuro [31], 10 plots of 10 m x 10 m (totalizing 0.1 hectare) were established in the forests surrounding each community. Plot locations were chosen by “preferential sampling” [32], where local experts identified the sites with the highest collection pressure (Fig 4). These local experts were invited to participate in the “walk in the wood” method [25] through the selected plots, where they named known and useful species. All the sampled species [15] were collected *in vivo* [24] and an image record was produced [33] for subsequent identification by comparison with CESJ Herbarium specimen [22].

The CPI was scored according to Table 3 and calculated using the formula below:

CPI=0.5(B)+0.5(RU)

B = Biological Value
RU = Risk of use

Where:

B = Dr x 10
Dr = (N/ni) x100
N = individuals of the x species
ni = individuals of all sampled species

RU=0.5(C)+0.5(U)×10

(C) Collection Risk **=** Points attributed per collected botanical parts
(U) Use-value = determined by the highest value between L and Div

**Table 3:** Scoring criteria used to determine conservation priority species. Adapted from [18].

	Criteria	Score
(Dr) Relative density	Occurrence between 0 and 1, then is considered too low	10
	Occurrence between 1.1 and 3.5, then is considered low	7
	Occurrence between 3.6 and 7, then is considered average	4
	Occurrence above 7	1
(C) Collection risk based on the botanical part collected	Removal of specimen, of descendants, excluding possibility of species perpetuation	10
	Removal of perennial structures without death, but actively influencing vegetative growth or flowering and perpetuation of species	7
	Ex: botanicals structures that fall naturally and periodically	
	Removal of permanent aerial parts without death and influencing only on vegetative growth and energy production	4
	Removal of transitory aerial parts without direct influence on species life cycle.	1
(L) Use location based on the reference frequency	For over than 20% of population, its use is considered high	10
	Between 10 and 20%, its use is considered moderately high	7
	Up to 10%, its use is considered moderately low	4
	Only mentioned in interviews	1
(Div) Diversity or plurality of use assigned to the species	For each use, add 1.42 to Div value	Up to 10

Analyzed species were categorized into three groups:

**Category 1** (species with score ≥ 85); they have conservation priority and should not be collected until appropriate precautions or for further conservation plans are implemented;

**Category 2** (species with score between 85 and 60); they are suitable for moderate collection;

**Category 3** (species with score ≤ 60); they are suitable for collection.

As another indicator of potential pressure on native species, the Use-Value Index (UVI) [25, 34] was calculated with the formula:

UVI=∑U/n

Where:

U = Number of mentioned uses of species X.
n = Total number of interviewees.

Lucena, Lucena [35] state that CPI is the most effective index to identify locally rare and impacted species, however, UVI can be additionally used to identify the most known and used species.

Finally, we classified species into their ecological succession stage by dividing them into three groups, according to the classification of Gandolfi, Leitão Filho [36] 1) Pioneer (species that develop in clearings, in forest edges or in the open, dependent on light and not occurring generally in the understory); 2) Early secondary (species that develop in small clearings in the understory under conditions of some shading and can also occur in areas of old clearings); 3) Late secondary (species that develop exclusively in the permanently shaded understory, including small or large tree species that develop slowly and may reach the canopy or are emerging; and 4) Climax (species that have slow growth, germinate and develop in the shade, and produce large seeds).

To compare our ethnobotanical indices to those in the literature, we searched for Ph.D. thesis and Master dissertations on Biblioteca Digital Brasileira de Teses e Dissertações (http://bdtd.ibict.br/vufind/) and for papers on Scientific Electronic Library Online (http://www.scielo.org/php/index.php) and Scopus (http://www.scopus.com/home.url) databases.

## Results and discussion

### Sociocultural characteristics

Based on experts at both communities, knowledge about local plants was predominantly among the older generation, with a mean of age of 58.7 ± 9.7 years in SSBV and 67.1 ± 3.9 in SB of the experts interviewed. Lima, Silva [37], Hanazaki, Tamashiro [38] and Galeano [23] have found similar results. This may indicate expertise takes many years, or that knowledge may be decreasing in the younger generations [23]. In our focus group discussions, it was noted that the decreasing isolation of these communities has resulted in changes in lifestyle, through the incorporation of urban elements into the local culture. This is also evidenced by the increase of households with TV and telephones and the education of 7 teenagers from SSBV and 5 from SB in Santos Dumont city. Participants in the focus group discussions also commented that young people are no longer interested in learning traditional knowledge.

In terms of gender, there were more female than male experts (Table 1), and all the women are medicinal plant experts and 4 of them are traditional cooks. All male experts are lumberjacks, bushman and builders—these knowledge categories are exclusive to men. These data demonstrate a social allocation of labor as the men are responsible for resource extraction from the forest and other jobs that require heavy labor, such as construction. Women are responsible for food preparation and health of their families. These results coincide with other studies of *Quilombola* communities [39, 40].

In terms of religion, 100% of the members of both communities are Catholic, demonstrating the great influence of Catholicism in historical and social process of the formation of Brazilian *Quilombola* communities’, as pointed out by Santos [41]. Historically this influence occurred due to the presence of large estates which were producers of coffee and milk, and where farmers imposed European culture on their slaves. This was confirmed through reports in both communities, that religion was one of the conditions imposed on them to keep the local peace. According to participants, in the case of SSBV, the most important historic milestone was the construction of the Church with the local farm owners help, in 1930 and the existence of a slave known as “Pai Tudo” (which translates to “father of everything”), who died in the same decade. He was considered a healer, spiritual healer, and sorcerer, who made magic for good and for evil and a local disseminator of religious and ethnobotanical knowledge. This highlights the religious syncretism and cultural changes that occurred as a result of imposed religious elements [42]. The local historic milestone in SB is similar to that of SSBV, where the Catholic Church was also constructed by farm owners.

### Ethnobotanical data

A total of 212 useful species were recorded from SSBV and 221 from SB. This included 105 and 96 native species from the Atlantic forest, respectively, totaling 139 native species (out of a total of 299) (Table 4). The substantial proportion of exotic species demonstrates the influence of diverse cultures and ethnic groups on plant knowledge formation at both communities.

**Table 4:** Two hundred and one native species cited as useful by the São Sebastião da Boa Vista (SSBV) and São Bento (SB) communities, in alphabetical order of botanical families, followed by vernacular name, species habit (Hab), use categories (Categ), plant part used, and voucher number.

Family	Scientific name (Family)	Vernacular name		Hab.	Use categories		Part		Voucher	
		SSBV	SB		SSBV	SB	SSBV	SB	SSBV	SB
Alismataceae	*Echinodorus grandiflorus* (Cham. & Schltdl.) Micheli	Chapéu de couro		Hb	M		Le		61724	
Amaranthaceae	*Alternanthera brasiliana* (L.) Kuntze	Amoxilina	Antibiótico de horta	Hb	M		Le		60495	
	*Dysphania ambrosioides* (L.) Mosyakin & Clemants	Santa Maria		Hb	M		Le		60489	
Anacardiaceae	*Anacardium occidentale* L.	Cajú		Ar	M		Le			
	*Schinus terebinthifolius* Radd	Aroeira		Ar	Fw	Fw; T	St			63310
Annonaceae	*Guatteria villosissima* A. St.-Hil.	Pindaíba		Ar	C; Fw	C; Fw	St			
	*Rollinia sylvatica* (A. St.-Hil.) Martius	Articum		Ar	Fw	C	St			
	*Xylopia sericea* A. St-Hill.	Andorinha		Ar	C		St			
	*Xylopia brasiliensis* Spreng.		Pau andorinha	Ar		T		St		
Apocynaceae	*Allamanda cathartica* L.		Mate	Sh		F		Le		
Araceae	*Xanthosoma sagittifolium* (L.) Schott.	Taioba		Hb	F		Le		62723	63279
Araucariaceae	*Araucaria angustifolia* (Bertol.) Kuntze	Pinheiro		Ar	F; T		Se; St			
Arecaceae	*Euterpe edulis* Mart.	Palmeira		Ar	F; T		St; Le			
Aristolochiaceae	*Aristolochia* sp.	Milihomi		Vi	M; R	M; R	E	Le		
Aspleniaceae	*Asplenium* sp.	Samambaiazinha		Hb	O				62737	
Begoniacea	*Begonia* sp^1^.		Azedinho	Hb		O				
Bignoniaceae	*Handroanthus chrysotrichus* (Mart. ex A. DC.) Mattos	Pau mulato	Ipê comum	Ar	T	Fw	St		62972	
	*Jacaranda caroba* (Vell.) DC.	Carobinha		Ar	Fw; T		St			63274
	*Pyrostegia venusta* (Ker Gawl.) Miers	Cipó São João		Vi	R	T	Le	E	63301	
	*Sparattosperma leucanthum* (Vell.) K. Schum.	Cinco folhas		Ar	M; Fw	M; Fw; R	St; Le			63309
	*Zeyheria tuberculosa* (Vell.) Bureau ex Verl.		Ipê graúdo	Ar		Fw	St			
Bixaceae	*Bixa orellana* L.	Urucum	Aricum	Ar	M; F	M	Se		62727	
Boraginaceae	*Tournefortia paniculata* Cham.	Marmelinho		Hb	M		Le; Fl	Fl		
Brassicaceae	*Brassica rapa* L.		Mostarda	Hb		F		Le		
Cactaceae	*Rhipsalis clavata* F.A.C. Weber		Chuveiro	Hb		O				
	*Schlumbergera truncata* (Haw.) Moran	Flor de maio		Hb	O	M; O		E	62743	
Campanulaceae	*Lobelia fistulosa* Vell.	Rabo de onça		Hb	M		Fl; St; Le			
Cannaceae	*Canna indica* L.	Bananeirinha	Imbirí de flor	Hb	O				62722	62997
Compositae	*Achyrocline satureioides* (Lam.)DC.	Marcela do campo		Hb	T		Fl		62794	
	*Ageratum conyzoides* (L.) L.	Erva de São João		Hb	M		Le; Ro	Le	60457	
	*Baccharis coridifolia* DC.	Alecrim do mato		Hb	R		Le		62790	
	*Baccharis pingraea* DC.		Santarina	Hb		M		Le		
	*Bidens pilosa* L.	Picão		Hb	M		Le		60532	63242
	*Cissampelos pareira* L.		Abuta branca	Vi		M		Le		
	*Eremanthus erythropappus* (DC.) MacLeish.	Candeia		Ar	C; Fw; T	C; T	St		62976	
	*Gochnatia polymorpha* (Less) Cabrera	Camará		Ar	C; T		St		62740	
	*Mikania glomerata* Spreng		Guaco	Hb		M		Le		
	*Mikania hirsutissima* var. *ursina* Baker	Cipó cabeludo		Vi	R	M	E	Le	62969	
	*Mikania* *cordifolia* (L.f.) Willd.	Cipó coração de Jesus		Vi	Fw		E		62775	
	*Piptocarpha axillaris* (Less.) Baker	Branda fogo		Ar	R; T		Le; St			
	*Solidago chilensis* Meyen	Arnica		Hb	M		Le		62459	
Davalliaceae	*Davallia* sp.	Samambaia		Hb	O				62749	
Dilleniaceae	*Davilla* *rugosa* Poir.	Cipó-caboclo		Vi	R	R; T	E		62791	63292
Dioscoreaceae	*Dioscorea* sp.	Inhame		Hb	M	F	Ro			
Euphorbiaceae	*Croton urucurana* Baill.	Adrago		Ar	C; Fw	Fw	St; Le	St	62793	62998
	*Manihot esculenta* Crantz	Mandioca		Hb	F		Ro		62721	62996
	*Maprounea guianensis* Aubl.	Santa Luzia		Ar	Fw; T		St			
	*Sapium glandulosum* (L.) Morong	Leiteira		Ar	M	C	St			
Hypericaceae	*Vismia brasiliensis* Choisy	Ruão		Ar	C; T	T	E	St	62783	
Lamiaceae	*Aegiphila sellowiana* Cham.	Papagaio		Ar	Fw		St		62984	63312
	*Aegiphila* sp.	Papagaio pequeno		Ar	Fw		St		62975	
	*Hyptidendron asperrimum* (Epling) Harley	Cinzeiro		Ar	Fw		St			
	*Peltodon radicans* Pohl.	Hortelã do mato		Hb	M		Le		60479	63258
	*Salvia splendens* Sellow ex Wied-Neuw.		Sirigaita	Hb		O				62992
Lauraceae	*Endlicheria* *paniculata* (Spreng.) J.F.Macbr.	Capoeira branca		Ar	Fw		St		62784	
	*Nectandra* *oppositifolia* Nees & Mart.	Canela branca	Canela	Ar	C; Fw		St		62782	
	*Ocotea odorifera* (Vell.) Rohwer		Sassafraz	Ar		Fw		St		
	*Ocotea puberula* (Rich.) Nees	Canela de rego		Ar	C; T		St			
	*Ocotea* sp.		Canela vermelha	Ar		Fw; T		St		
Leguminosae	*Andira anthelmia* (Vell.) J.F.Macbr.	Limpeza do mundo		Ar	R		St; Le		61713	
	*Dalbergia hortensis* Heringer & al.	Endireita mundo		Ar	M; C; R; T	T	St; Le; Fl		65415	65390
	*Machaerium isadelphum* (E.Mey.) Standl.	Muchoco		Ar	T		St		62731	
	*Machaerium nyctitans* Benth (Vell.)	Bico de pato		Ar	Fw	T	St		63306	63265
	*Machaerium* sp.	Angú seco		Ar	T		St			
	*Machaerium villosum* Vogel	Jacarandá roxo		Ar	T		St			
	*Machaerium dimorphandrum *Hoehne		Angú-seco	Ar		T		St		
	*Machaerium scleroxylon* Tul.		Caveiúna	Ar		C; Fw		St		
	*Piptadenia* *gonoacantha* (Mart.) J.F.Macbr.	Pau jacaré, Jacaré		Ar	C; Fw; T	Fw; T	St		62789	63287
	*Platypodium* *elegans* Vogel	Jacarandá branco		Ar	T		St		62778	
	*Senna* *macranthera* (Collad.) H.S.Irwin & Barneby	Pau de cachimbo		Ar	C; T	O; T	St		62751	62989
	*Stryphnodendron polyphyllum* Mart.	Barbatimão		Ar	M; Fw; R	M; Fw; R; T	St; Ba	St; Ba; Le	60520	
Lygodiaceae	*Lygodium volubile* SW.	Segue caminho	Abre caminho	Hb	O; R	R	E		62738	63291
Lythraceae	*Cuphea* sp.		Vassoura canela de saracura	Hb		R; T		E		63302
Cyatheaceae	*Cyathea sp*.	Samambaiaçú		Ar	M; O; T	T	E	St; Le	62776	63280
	*Cyathea* sp.^1^		Samambaia	Hb		O				63025
Malpighiaceae	*Malpighia glabra* L.	Acerola		Ar	M; F	F	Fr			
Malvaceae	*Luehea divaricata* Mart.	Açoita cavalo		Ar	M; R		Le		62980	
	*Pseudobombax* sp.		Imbíra	Ar		F; T		Fr; Se		
	*Sida* *acuta* Burm.f.	Vassoura babosa		Hb	M; O; R; T		E		62745	
	*Sida rhombifolia* L.	Vassoura		Hb	T		E			63002
Melastomataceae	*Leandra nianga* Cogn.		Quaresminha	Ar		O; Fw		E		
	*Leandra sericea* DC.		Quaresmeirinha	Ar		T		St		
	*Leandra sp*.	Quaresminha		Ar	Fw		St			
	*Miconia albicans* (Sw.) Steud.		Quaresminha	Ar		O; Fw		E		
	*Miconia cinnamomifolia* (DC.) Naudin	Muricí		Ar	C; Fw	C; Fw; T	St			
	*Miconia sp*.		Zumbi	Ar		C; Fw; T		St		
	*Miconia sp*^1^.		Murici cabeça de boi	Ar		C; Fw		St		
	*Miconia sp*^2^.		Zumbi	Ar		Fw; T		St		
	*Miconia* *cubatanensis* Hoehne	Zumbi	Carvãozin	Ar	Fw	T	St		62785	63257
	*Tibouchina* *granulosa* (Desr.) Cogn.	Chorão		Ar	C; Fw; T	T	St		62788	
	*Tibouchina semidecandra* (Mart. & Schrank ex DC.) Cogn.		Quaresminha	Ar		O				
Meliaceae	*Cabralea canjerana* (Vell.) Mart	Tento		Ar	T		St			
	*Cedrela fissilis* Vell.	Cedro		Ar	C; T	C	St			
Myrtaceae	*Eugenia uniflora* L.	Pitanga		Ar	F		Fr		63269	63271
	*Myrcia guianensis* (Aubl.) DC.	Goiabinha		Ar	F		Fr			
	*Myrcia perforata* O.Berg		Gumirim	Ar		C; Fw; T		St		
	*Myrcia splendens* (Sw.) DC.	Gumirim		Ar	C; Fw	C; Fw; T	St		63266	
	*Psidium cattleianum* Afzel. ex Sabine	Araça miúdo		Ar	M; F		Fr		62781	
	*Psidium guineense* SW.	Goiaba		Ar	F		Fr		62757	
Nephrolepidaceae	*Nephrolepis* sp.	Samambaia		Hb	O				62746	
Passifloraceae	*Passiflora edulis* Sims	Maracujá		Vi	F; M		Fr			
	*Passiflora* sp.	Maracujá		Vi	F		Fr		62786	
Phyllanthaceae	*Phyllanthus tenellus* Roxb.	Quebra pedra		Hb	M		Le	E	60531	63243
Piperaceae	*Peperomia* *glabella* (Sw.) A.Dietr.	Rabo de rato		Hb	O				62761	
	*Piper arboreum* Aubl		Jarabandí grande	Ar		M		Ro		63004
	*Piper miquelianum* C. DC.	Jarabandí		Sh	M		Ro			63284
	*Piper* sp.		Jarabandí graúdo	Ar		M		Ro		63299
	*Piper umbellatum* L.	Capeva		Hb	M; R		Le	E	62970	63009
Plantaginaceae	*Scoparia dulcis* L.	Vassoura de Nossa Senhora		Hb	T	M	E	Le		
Poaceae	*Imperata brasiliensis* Trin.	Sapê		Hb	C; T		E			
	*Merostachys* sp.	Taquarinha		Hb		T		St		
	*Merostachys* sp^1^.	Taquara		Hb	O; C; Fw; T	C	St			
Polygonaceae	*Polygala paniculata* L.		Vassourinha de benzer	Hb		R		Le		
Polypodiaceae	*Phlebodium decumanum* (Willd.)J.Sm.	Samambaia chorona		Hb	O				62733	
Primulaceae	*Myrsine guianensis* (Aubl.) Kuntze	Pororoca		Ar	C; Fw	Fw	St		62773	62994
Pteridaceae	*Adiantum* sp.	Avenca		Hb	M; O				62735	
Rosaceae	*Rubus rosifolius* SM	Amora do mato		Hb	F		Fr		62772	62986
Rubiaceae	*Galianthe brasiliensis* (Spreng.) E.L. Cabral & Bacigalupo		Vassoura cabelo de nega	Hb		T		E		
	*Richardia brasiliensis* Gomes	Puaia		Hb	M		Le		62460	
Rutaceae	*Zanthoxylum rhoifolium* Lam.	Mamica de porca		Ar	Fw	C; Fw; T	St			
Salicaceae	*Casearia arborea* (Rich.) Urb.	Canela de veado		Ar	T		St		63268	
	*Casearia lasiophylla* Eichler		Canela de veado	Ar		O; T		St		63307
	*Casearia sylvestris* Sw.	Erva lagarto		Ar	M	M; R	Le		60455	63241
Sapindaceae	*Cupania ludowigii* Somner & Ferrucci	Camboatá		Ar	C		St		62977	
	*Cupania vernalis* Cambess.		Canjerona	Ar		T		St		
Scrophulariaceae	*Buddleja stachyoides* Cham. & Schltdl.	Barbaço		Hb	M		Le		60491	63276
Siparunaceae	*Siparuna* *brasiliensis* (Spreng.) A. DC.	Limãozinho		Ar	R		Le		62979	
	*Siparuna guianensis* Aubl.	Negra mina		Ar	R	M; R	Le		63008	
Solanaceae	*Acnistus arborescens* (L.) Schltdl.		Maria neira	Ar		Fw; R		St; Le		63273
	*Aureliana tomentosa* Sendtn.		Pau canjenga	Ar		R		E		
	*Capsicum baccatum* var. *praetermissum* (Heiser & P.G.Sm.) Hunz.	Pimenta		Hb	F		Fr		62744	
	*Solanum americanum* Mill.	Erva moura		Hb	M		Le		60513	63262
	*Solanum cernuum* Vell.	Panacéia		Sh	M		Le		60534	
	*Solanum lycocarpum* A. St.-Hil.	Fruta de lobo		Sh	M	F	Fr		60473	63012
	*Solanum paniculatum* L.		Jurubeba	Hb		M		Le		
Urticaceae	*Cecropia glaziovii* Snethl.	Imbaúba		Ar	C; T		St		62787	63267
Verbenaceae	*Duranta erecta* L.	Pingo de ouro		Sh	M; O	O	E		62750	62990
	*Lippia alba* (Mill.) N.E. Br. ex Britton & P. Wilson	Erva cidreira		Sh	M		Le		60466	
Zingiberaceae	*Hedychium* *coronarium* J.Koenig	Imbirí		Hb	O				62777	

(Ar) = arboreal, (Sh) = shrub, (Hb) = herb, (Vi) = vine, (F) = food, (C) = construction, (Fw) = fuelwood, (M) = medicinal, (O) = ornamental, (R) = ritualistic, (T) = technological, (Le) = leaves, (Fl) = flowers, (Fr) = fruits, (Ba) = bark, (St) = stem, (Se) = seeds, (Ro) = roots, (E) = entire.

In general, those plants used in the two communities were used in the same ways in both places. However, a few species had different uses, such as *Dalbergia hortensis* (used for medicinal, construction, ritualistic and technological uses in SSBV and only technological uses in SB) and *Merostachys* sp^1^. (employed in ornamental, construction, fuelwood and technological uses in SSBV and only used for construction in SB). A possible explanation is that they were influenced by different farmers in their respective areas, which possibly resulted in different knowledge about the same plants. Although *Quilombolas* knowledge includes knowledge brought from Africa, it also includes knowledge learned from Amerindians and Europeans living in Brazil. This influence can be observed in the vernacular names of plants, which are distinct in many cases between the two communities (Table 4).

**Fig 5:**
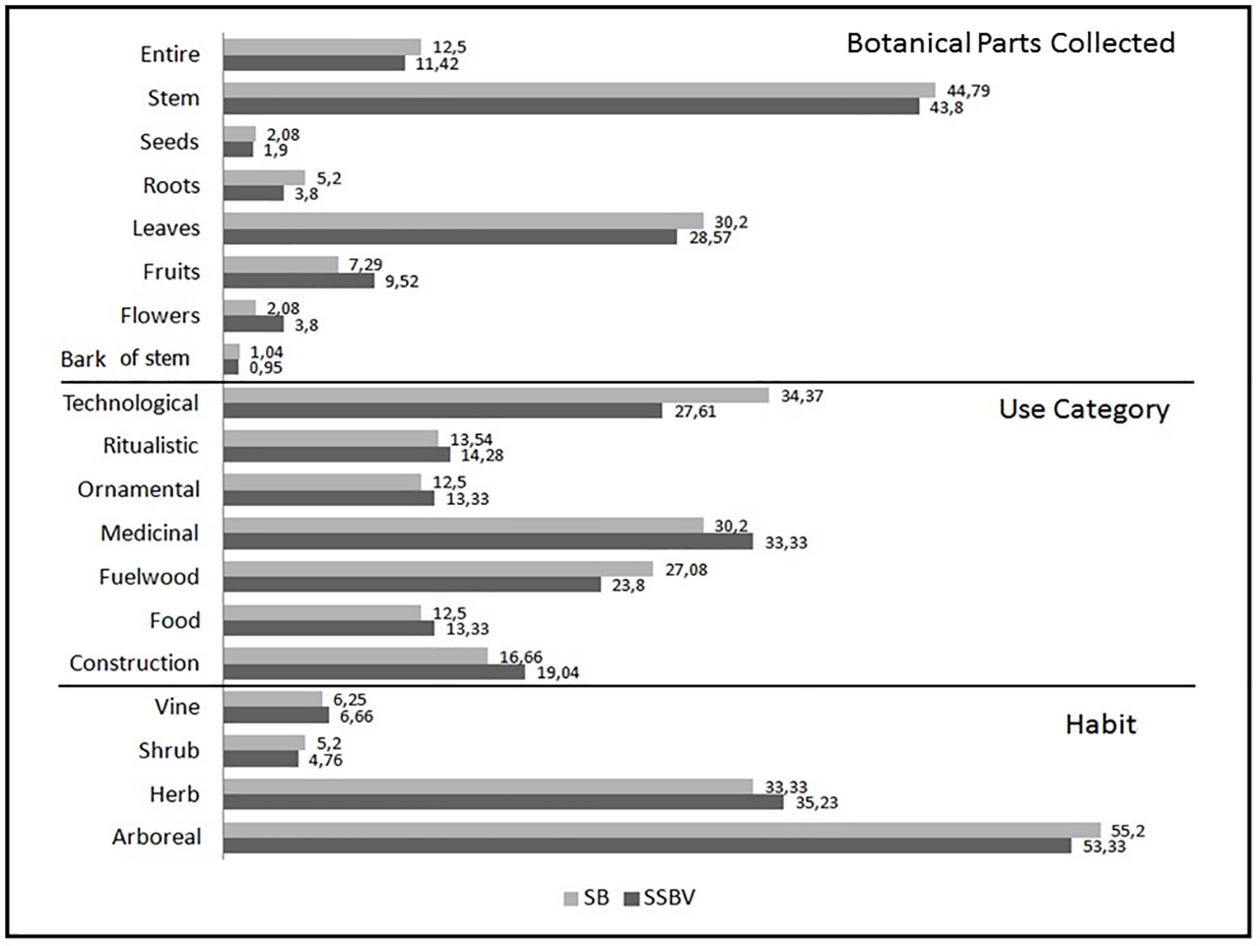
Comparison of plant parts collected, use category and habit of native species of ethnobotanical importance cited in the interviews with local experts in São Sebastião da Boa Vista (SSBV) and São Bento (SB). Values represent percentages (%) of total species reported.

Medicinal and technological uses were the most important uses in both communities (Fig 5). The predominance of plants used for medicinal purposes was also described for other *Quilombolas* communities, including Campinho da Independência in Paraty/RJ, Brazil [16] and in Espírito Santo state, Brazil [15], both in areas of Atlantic Rainforest. Hanazaki, Souza [43] similarly described the main use of plants for medicinal purposes for rural communities in the Boundaries of Carlos Botelho State Park in São Paulo, Brazil. In this study construction/technological uses included construction of houses and furniture, manufacturing of handles, canoes, fence posts and wooden wagons. This is similar to another Atlantic forest community (Rio Formoso/PE, Brazil) where technology and medicine were identified as the two most important use categories [44].

We found that herbaceous plants are predominant among medicinal species, and that leaves are the plant part most commonly collected from herbaceous species. Trees were mostly employed for technological uses and therefore stems were the plant part most commonly used. In Rio Formoso, the plant part most frequently collected part was wood (78.5%), followed by fruit, bark, resin, inner bark, seed, leaf, and flowers [44]. Albuquerque and Andrade [45], Oliveira, Lins Neto [46] and Meyer, Quadros [47] showed the predominant use of stems and trees in the Caatinga; however, it is important to note that this biome has different characteristics to the Atlantic Rainforest, as it is much drier.

The Shannon-Wiener biological diversity index and Equitability index were 5.14 and 0.96 respectively for SSBV and 5.20 and 0.96 for SB. These are considered high according to [29] and as compared to other studies in Brazil (Table 5). These values may indicate homogeneity of ethnobotanical knowledge. However, Meyer, Quadros [47] state that high values can also demonstrate a common ethnobotanical knowledge origin of plant knowledge. This is consistent with the fact that among the 63 species that were used in both communities, 42 species have the same vernacular name (Table 4). The high evenness may also be a result of the fact that only experts were interviewed in each community. However, our value for the diversity of ethnobotanical knowledge is similar to that found for another *Quilombolas* community (Table 5). The high diversity of knowledge could potentially be a result of the fact that *Quilombolas* ethnobotanical knowledge includes a combination of African, Amerindian and European knowledge of plants.

**Table 5:** Comparison of ethnobotanical diversity indices compiled from studies of traditional communities in Brazil.

City/Brazilian state	Reference	Type of community	Biome	Comprehensiveness	EI	H' B.e	N° sp.	N° infor.	N° cit.
Barcarena/ PA	[48]	Rural	Amazon	Medicinal	0.94	5.07	220	17	365
Xapurí/ AC	[49]	Rural	Amazon	All useful plant species	0.97	4.80	145	14	1284
Ubatuba/ SP	[38]	Coastal caiçara fisher-men	Atlantic Rainforest	All useful plant species	-	4.57	162	57	541
Guaraqueçaba/PR	[37]	Rural	Atlantic Rainforest	All useful plant species	-	5,48	445	90	3400
Santo Antônio do Leverger/ MT	[50]	Rural	Pantanal	Medicinal	0,94	5,09	228	48	938
Arraial do Cabo/ RJ	[33]	Coastal caiçara fisher-men	Atlantic Rainforest	All useful plant species	-	4,1	68	15	444
Ingaí/ MG	[51]	Urban	Atlantic Rainforest	All useful plant species	0,76	4,84	178	17	-
Silva Jardim/ RJ	[52]	Rural	Atlantic Rainforest	All useful plant species	-	5,07	209	19	548
Itacaré/ BA	[53]	Rural	Atlantic Rainforest	Medicinal	0,92	4,21	98	26	379
Mogi Mirim/ SP	[54]	Urban	Atlantic Rainforest / Cerrado	Medicinal	0,87	4,07	107	50	516
Rio Negro/ AM	[55]	Caboclo river-dwellers	Amazon	All useful plant species	-	4,71	425	33	180
Rio Negro/ AM	[55]	Caboclo river-dwellers	Amazon	All useful plant species	-	4,75	632	48	194
Santa Leopoldina/ ES	[15]	Quilombolas	Atlantic Rainforest	All useful plant species	-	5,12	188	11	-
Anchieta/ SC	[56]	Rural	Atlantic Rainforest	All useful plant species	0,98	4,31	101	78	776
Poxim-Açu/ SE	[57]	Rural	Atlantic Rainforest	All useful plant species	0,73	3,9	126	31	-
Anastácio/ MS	[58]	Rural	Cerrado	Medicinal	0,94	5,03	209	35	-
Ascurra/ SC	[47]	Rural	Atlantic Rainforest	Medicinal	0,92	4,23	109	42	314
Paraty/ RJ	[59]	Coastal caiçara fisher-men	Atlantic Rainforest	All useful plant species	-	5,03	190	12	1341
Viçosa/ MG	[60]	Rural	Atlantic Rainforest	Non-conventional food plants	0.93	1.65	59	20	389
Paracambi/RJ	[61]	Municipal Natural Park	Atlantic Rainforest	Random sampling	0.88	4.7	210	-	749
**São Sebastião da Boa Vista/ MG**	**Present study**	**Quilombolas**	**Atlantic Rainforest**	**All useful plant species**	**0,96**	**5,14**	**212**	**7**	**530**
**São Bento/ MG**	**Present study**	**Quilombolas**	**Atlantic Rainforest**	**All useful plant species**	**0,96**	**5,21**	**221**	**6**	**476**

(EI) = Equitability index, (H’ B.e) = Shannon index base, (N° sp.) = Number of cited species, (N° infor.) = Number of informants, (N° citat.) = Number of citations.

The forest species used in both communities are, in general, categorized as low risk based on international (International Union for the Conservation of Nature (IUCN), and national (Biodiversitas and Ministério do Meio Ambiente—MMA) assessments (Table 6). At SSBV, *A*. *angustifolia* and *E*. *edulis* are classified as “in danger” according to Biodiversitas and “endangered” according to MMA and *M*. *villosum* is “vulnerable” according to IUCN. At SB, only *O*. *odorifera* is classified as “in danger” according to Biodiversitas and Endangered according to MMA.

**Table 6:** Native forest species cited as useful by the study communities (SSBV and SB), in alphabetical order of botanical species, followed by conservation priority, category, use-value, cultural significance index, risk category.

Species	Conservation Priority				Use Value		Cultural Significance Index		Risk Category
	Score		Category						
	SSBV	SB	SSBV	SB	SSBV	SB	SSBV	SB	
*Acnistus arborescens* (L.) Schltdl.		85		1		0.32		0.32	
*Aegiphila sellowiana* Cham.	100	100	1	1	0.42	0.82	0.86	1.66	
*Aegiphila* sp.	92.5		1		0.14		0.14		
*Andira anthelmia* (Vell.) J.F.Macbr.	85		1		0.85		1.72		
*Araucaria angustifolia* (Bertol.) Kuntze	92.5		1		0.14		0.14		ID, ED
*Aristolochia* sp.	92.5	100	1	1	0.71	0.67	1.71	1.5	
*Aureliana tomentosa* Sendtn.		75		2		0.32		0.96	
*Cabralea canjerana* (Vell.) Mart	62.5	67.5	2	2	0.14	0.17	0.14	0.16	
*Casearia arborea* (Rich.) Urb.	92.5		1		0.14		0.14		
*Casearia lasiophylla* Eichler		85		1		0.32		1.32	
*Casearia sylvestris* Sw.	85	62.5	1	2	0.42	0.67	1.72	2	
*Cecropia glaziovii* Snethl.	100	70	1	2	0.42	0.67	0.84	3.32	
*Cedrela fissilis* Vell.	100	100	1	1	0.85	0.5	2.85	1	
*Cissampelos pareira* L.		100		1		0.17		0.32	
*Croton urucurana* Baill.	100	100	1	1	0.85	1	2.84	2	
*Cupania ludowigii* Somner & Ferrucci	100		1		0.28		0.56		
*Cupania vernalis* Cambess.		100		1		0.32		0.66	
*Cuphea* sp.	62.5	75	2	2	0.42	0.32	0.28	1.32	
*Cyathea* sp.		92.5		1		0.17		0.16	
*Dalbergia hortensis* Heringer & al.	100	92.5	1	1	2.14	0.17	26	1	
*Davilla rugosa* Poir.	70	70	2	2	0.42	0.32	0.86	0.66	
*Endlicheria paniculata* (Spreng.) J.F.Macbr.	92.5	100	1	1	0.14	0.32	0.14	0.66	
*Eremanthus erythropappus* (DC.) MacLeish.	70	100	2	1	1	0.5	6.88	2	
*Euterpe edulis* Mart.	77.5		2		0.28		0.28		ID, ED
*Gochnatia polymorpha* (Less) Cabrera	92.5		1		0.28		0.14		
*Guatteria villosissima* A. St.-Hil.	85	100	1	1	0.57	0.67	0.56	0.66	
*Handroanthus chrysotrichus* (Mart. ex DC.) Mattos	100	77.5	1	2	0.42	0.17	0.86	0.16	
*Hyptidendron asperrimum* (Spreng.) Harley	92.5		1		0.14		0.14		
*Jacaranda caroba* (Vell.) DC.		77.5		2		0.32		0.32	
*Leandra nianga* Cogn.		92.5		1		0.32		0.16	
*Leandra sericea* DC.		100		1		0.32		0.16	
*Leandra* sp.	70		2		0.42		0.86		
*Lobelia fistulosa* Vell.	77.5		2		0.14		0.14		
*Luehea divaricata* Mart.	85		1		0.57		1.12		
*Lygodium volubile* Sw.	85	100	1	1	0.28	0.32	3.36	0.48	
*Machaerium sp*.	70		2		0.28		3.36		
*Machaerium isadelphum* (E.Mey.) Standl.	77.5		2		0.14		0.28		
*Machaerium nyctitans* (Vell.) Benth.		85		1		0.32		0.66	
*Machaerium villosum* Vogel	92.5		1		0.14		0.28		V
*Machaerium dimorphandrum Hoehne*		85		1		0.17		0.32	
*Machaerium scleroxylon *Tul.		100		1		0.5		0.99	
*Maprounea guianensis* Aubl.	55		3		0.85		1.72		
*Merostachys* sp.	85	70	1	2	1	0.17	2.28	0.16	
*Miconia albicans* (Sw.) Steud.		92.5		1		0.32		0.16	
*Miconia cinnamomifolia* (DC.) Naudin	85	70	1	2	0.28	0.17	1.12	0.16	
*Miconia cubatanensis* Hoene	70	85	2	1	0.42	0.5	0.56	1	
*Miconia* sp.		77.5		2		0.32		0.16	
*Miconia* sp.^1^		100		1		0.5		0.16	
*Mikania cordifolia* (L.f.) Willd	77.5		2		0.14		0.14		
*Mikania hirsutissima* var. *ursina* Baker	77.5	55	2	3	0.14	0.32	0.14	0.16	
*Myrcia guianensis* (Aubl.) DC.	70		2		0.14		0.14		
*Myrcia perforata* O.Berg		62.5		2		0.5		0.16	
*Myrcia splendens* (Sw.) DC.	55	100	3	1	0.71	1	0.57	3	
*Myrsine guianensis* (Aubl.) Kuntze		85		1		0.32		0.66	
*Nectandra oppositifolia* Nees & Mart.	100	85	1	1	1	1	1.14	0.32	
*Ocotea odorifera* (Vell.) Rohwer		77.5		2		0.17		0.32	VU, ED
*Ocotea* sp.		92.5		1		0.17		0.32	
*Ocotea puberula* (Rich.) Nees	100		1		0.28		0.56		
*Passiflora* sp.	70	70	2	2	0.28	0.17	0.14	0.16	
*Piper arboreum* Aubl.		77.5		2		0.17		0.16	
*Piper miquelianum* C. DC.	85		1		0.28		1.12		
*Piper* sp.		92.5		1		0.17		2.5	
*Piper umbellatum* L.	77.5	92.5	2	1	0.14	0.82	0.56	0.16	
*Piptadenia gonoacantha* (Mart.) J.F.Macbr.	55	70	3	2	1	1	4.3	3.32	
*Piptocarpha axillaris* (Less.) Baker	77.5		2		0.14		1.12		
*Platypodium elegans* Vogel	85		1		0.28		0.56		
*Pseudobombax* sp.		92.5		1		0.32		0.16	
*Psidium cattleianum* Afzel. ex Sabine	77.5		2		0.28		0.56		
*Psidium guineense* SW.	77.5		2		0.28		0.56		
*Pyrostegia venusta* (Ker Gawl.) Miers	77.5	85	2	1	0.14	0.32	0.14	0.66	
*Rollinia sylvatica* (A. St.-Hil.) Martius	100	92.5	1	1	0.28	0.17	0.56	0.16	
*Sapium glandulosum* (L.) Morong	85	77.5	1	2	0.28	0.17	0.56	0.16	
*Schinus terebinthifolia* Raddi	92.5	55	1	3	0.14	0.32	0.14	0.66	
*Senna macranthera* (Collad.) H. S. Irwin & Barneby	100	70	1	2	0.42	0.32	1.12	1.98	
*Siparuna brasiliensis* (Spreng) A. DC.	77.5	55	2	3	0.42	0.67	0.86	1	
*Siparuna guianensis* Aubl.	70	55	2	3	0.28	0.67	1.12	1	
*Sparattosperma leucanthum* (Vell.) K. Schum.	85	70	1	2	0.71	1	4.26	3.32	
*Stryphnodendron polyphyllum* Mart.		70		2		0.17		2	
*Tibouchina granulosa* (Desr.) Cogn.	70	77.5	2	2	1	0.32	6.02	1.32	
*Tibouchina semidecandra* (Mart. & Schrank ex DC.) Cogn.		47.5		3		0.17		0.16	
*Vismia brasiliensis* Choisy.	55	47.5	3	3	0.57	0.17	1.72	0.16	
*Xylopia sericea* A. St-Hill.	92.5		1		0.14		0.14		
*Xylopia brasiliensis* Spreng.		77.5		2		0.67		0.16	
*Zanthoxylum rhoifolium* Lam.	85	77.5	1	2	0.42	0.5	0.86	0.16	
*Zeyheria tuberculosa* (Vell.) Bureau ex Verl.		92.5		1		0.17		0.16	

(ID) = In danger by Biodiversitas, (ED) = Endangered by Ministry of the Environment, (V) = Vulnerable by International Union for Conservation of Nature, (VU) = Vulnerable by Biodiversitas. Category 1 (Cat 1)–species with score ≥ 85 are of conservation priority and should not be collected if appropriate precautions are not taken; Category 2 (Cat 2)–species with score between 85 and 60 can be moderately collected; Category 3 (Cat 3)–species with score ≤ 60 are suitable for collection.

Unfortunately, locally these species appear to be at much higher risk. The results of our conservation priority index show that, of the 59 species at SSBV in Table 6, 56% are classified in Category 1 (highest risk), 37% of Category 2 and 7% in Category 3. Among the 61 forest species of SB, 52% were classified in Category 1, 38% in Category 2 and 10% in Category 3. This indicates that more than 50% of the forest species are under threat and would benefit from conservation plans. Although the *Quilombolas* do not harvest plants for commercial purposes, some of their species have high economic value. Some species in the highest category for conservation priority such as *Ocotea odorifera* and *Machaerium scleroxylon*, are used for the production of luxury furniture production and in civil construction [62]. This tends to attract harvesting by people from outside of the communities. This emphasizes the need for a management plan for the biodiversity of the region.

Another complicating factor is that among species with highest conservation priority (Category 1), 14 (23.7%) and nine (28.1%) were also of high cultural significance (values above 1) in SSBV and SB, respectively. These results show that some of the most culturally important species are also among the most vulnerable locally. Species with both high use value indices and CSI included *Dalbergia hortensis* (26/2.14), *Eremanthus erythropappus* (6.88/1) and *Tibouchina granulosa* (6.02/1) at SSBV, and *Piptadenia gonoacantha* (3.32/1), *Sparattosperma leucanthum* (3.32/1) and *Cecropia glaziovii* (3.32/0.67) at SB.

**Fig 6:**
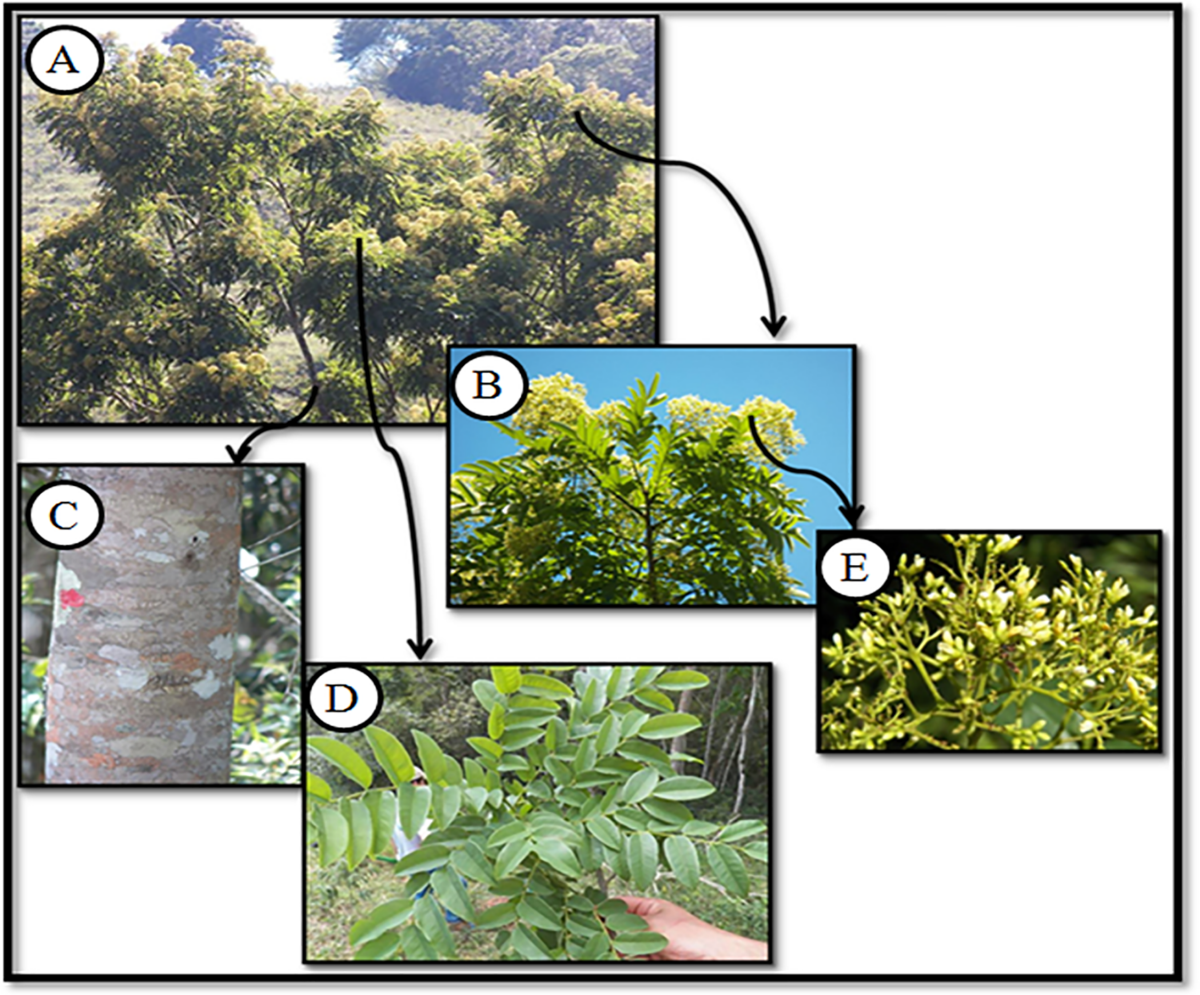
*Dalbergia hortensis* Heringer & al. (A) = Apical region with inflorescences, (B and E) = Detailed inflorescences, (C) = Detailed Stalk, (D) = detailed leaves.

By far the species with the highest cultural significance index (CSI) was *Dalbergia hortensis* (CSI = 26 in SSBV) (Fig 6). The use of this species in SSBV was disseminated by “Pai Tudo”. In SB, Pai Tudo was also mentioned, but only *Aureliana tomentosa* was identified to be learned from him, and it does not have a high CSI (0.96). Knowledge related to this species is considered a cultural secret [63] since it was reported by the leader of SB as having a ritualistic power capable of causing harmful effects even to oneself if handled by a non-expert.

### Forest succession stages

**Fig 7:**
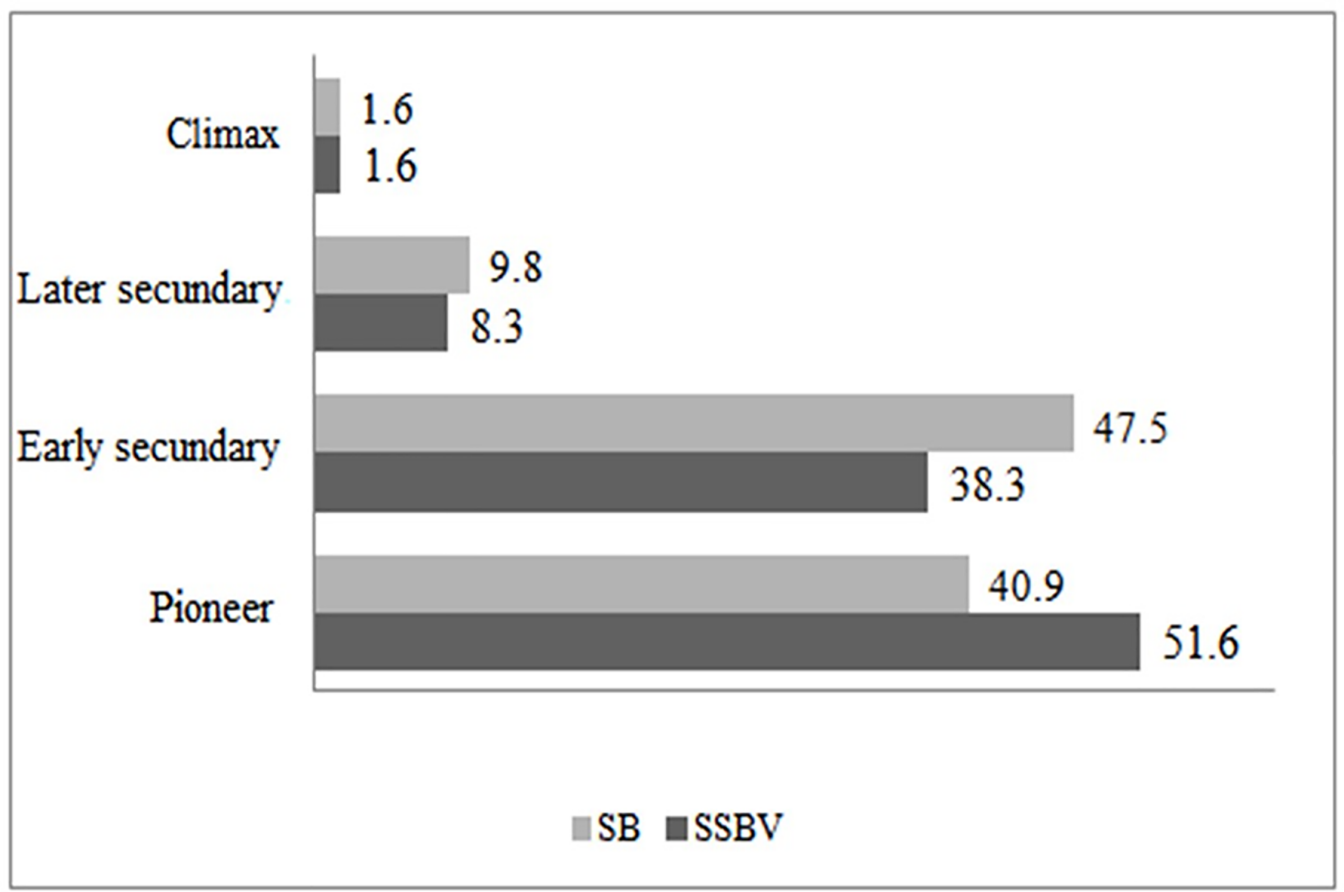
Successional stages of native forest trees in São Sebastião da Boa Vista (SSBV) and São Bento (SB). Results are expressed in percentage (%).

Of the native species identified, 85 were forest trees, including 59 in SSBV and 61 in SB. Thirty-five were common to both communities. Pioneer species predominate in SSBV, while early secondary predominates in SB (Fig 7), demonstrating that the forest SSBV is in an earlier stage of regeneration than SB. This may indicate that the SB forests are relatively better preserved than those of SSBV, however, further phytosociological study is needed.

According to interviews with local experts in both communities, local forests have sharply declined in the last 50 years due to an increase in grazing lands. According to reports of SSBV, the increase in agricultural activities since the 1960s and the onset of charcoal factories in the 1970s have consumed forest native trees as the main fuel stock. In SB it was reported that historically farmer owners used to lend part of their land to *Quilombolas* in exchange of work on crop and cattle ranches. *Quilombolas* were required to cut down part of their forests to increase land for agriculture and for cattle grazing. Therefore, in the cases of species like *A*. *angustifolia* and *M*. *villosum*, where the high use coincides with high conservation threat, it is likely not just harvest but more importantly habitat destruction that is causing decline.

## Conclusion

Our interviews showed that together, the two *Quilombolas* communities of SB and SSVB use 201 native species, and have ethnobotanical knowledge diversity indices of over 5.0—values that are higher than those reported for other traditional groups in Brazil. These data illustrate the rich ethnobotanical knowledge and heritage of the communities. However, our results also suggest that more than 50% of local useful species in both communities (those ranked in Category 1 for conservation priority) may be at risk if there are no plans for the management and replanting of them. Of these plants, *Dalbergia hortensis* is a special conservation priority because of its great cultural significance. Other species such *Sparattosperma leucanthum*, *Lygodium volubile* in SSBV, *Cecropia glaziovii* in SB, and *Croton urucurana* in both communities rank high for cultural significance and conservation priority. Based on our results, the development of a sustainable management plan that considers local knowledge about management and use of plants is essential. Developing programs to increase populations of those species at risk, including agroforestry programs can help meet the needs of producing culturally important species and of biological conservation. It is urgent that the government demarcate *Quilombolas* land for cultural maintenance, quality of life and preservation of nature.

## Supporting information

S1 Appendix Permission to the conduction of this study emitted by Instituto do Patrimônio Histórico e Artístico Nacional (IPHAN). (PDF)
