# Supplementary material for: Local ecological knowledge and its relationship with biodiversity conservation among two Quilombola groups living in the Atlantic Rainforest, Brazil
Source: PLoS One. 2017 Nov 28;12(11):e0187599. doi: 10.1371/journal.pone.0187599 (PMC5705149; doi:10.1371/journal.pone.0187599)
Supplement: S1 Appendix — (PDF) [file pone.0187599.s001.pdf]

EXTRATO DE INEXIGIBILIDADE DE LICITAÇÃO Nº 1012/2013 - UASG 403201

Nº Processo: 01530000178201358 . Objeto: Contratação da artista Betriz Goes do Espirito Santo, para apresentação do show musical que ocorrerá no dia 17 de fevereiro do corrente ano,no Espaço Brasil em Lisboa/Portugal, no âmbito das comemorações do Ano Brasil-Portugal. Total de Itens Licitados: 00001 . Fundamento Legal: Art. 25º, Inciso III da Lei nº 8.666 de 21/06/1993. . Justificativa: Conforme Processo: 01530.000178/2013-58 Declaração de Inexigibilidade em 06/02/2013 . ANA AMELIA DE CARVALHO VELLOSO . Coordenadora do Gabinete da Presidência . Ratificação em 06/02/2013 . ANTONIO CARLOS GRASSI . Presidente da Funarte . Valor Global: R$ 10.000,00 . CNPJ CONTRATADA : 01.472.044/0001-87 CARLOS HA MILTON MARTINS FELTRIN - ME.

(SIDEC - 07/02/2013) 403201-40402-2012NE800025

EXTRATO DE INEXIGIBILIDADE DE LICITAÇÃO Nº 1013/2013 - UASG 403201

Nº Processo: 01530000171201336 . Objeto: Contratação do artista Jair Rodrigues Melo de Oliveira "Jair de Oliveira", para apresentação do show musical que ocorrerá no dia 21 de fevereiro do corrente ano,no Espaço Brasil em Lisboa/Portugal, no âmbito das comemorações do Ano Brasil-Portugal. Total de Itens Licitados: 00001 . Fundamento Legal: Art. 25º, Inciso III da Lei nº 8.666 de 21/06/1993. . Justificativa: Conforme Processo: 01530.000171/2013-36 Declaração de Inexigibilidade em 06/02/2013 . ANA AMELIA DE CARVALHO VELLOSO . Coordenadora do Gabinete da Presidência . Ratificação em 06/02/2013 . ANTONIO CARLOS GRASSI . Presidente da Funarte . Valor Global: R$ 10.000,00 . CNPJ CONTRATADA : 08.741.133/0001-20 RCS PRODU COES ARTISTICAS E CINEMATOGRAFICAS LTDA - ME.

(SIDEC - 07/02/2013) 403201-40402-2012NE800025

EXTRATO DE INEXIGIBILIDADE DE LICITAÇÃO Nº 1014/2013 - UASG 403201

Nº Processo: 01530000172201381 . Objeto: Contratação do artista Luís de França Guilherme de Queiroga Filho "Lula Queiroga", para apresentação do show musical que ocorrerá no dia 23 de fevereiro do corrente ano,no Espaço Brasil em Lisboa/Portugal, no âmbito das comemorações do Ano Brasil-Portugal. Total de Itens Licitados: 00001 . Fundamento Legal: Art. 25º, Inciso III da Lei nº 8.666 de 21/06/1993. . Justificativa: Conforme Processo: 01530.000172/2013-81 Declaração de Inexigibilidade em 06/02/2013 . ANA AMELIA DE CARVALHO VELLOSO . Coordenadora do Gabinete da Presidência . Ratificação em 06/02/2013 . ANTONIO CARLOS GRASSI . Presidente da Funarte . Valor Global: R$ 10.000,00 . CNPJ CONTRATADA : 01.374.871/0001-38 LUNI PROD UCOES LTDA - ME.

(SIDEC - 07/02/2013) 403201-40402-2012NE800025

EXTRATO DE INEXIGIBILIDADE DE LICITAÇÃO Nº 1015/2013 - UASG 403201

Nº Processo: 01530000169201367 . Objeto: Contratação da cantora Jussara Maria Silveira Ferreira, em artes Jussara Silveira, para realizar uma apresentação,no Espaço Brasil, no âmbito do evento Ano do Brasil em Portugal, na cidade de Lisboa/Portugal, no dia 15 de fevereiro de 2013. Total de Itens Licitados: 00001 . Fundamento Legal: Art. 25º, Inciso III da Lei nº 8.666 de 21/06/1993. . Justificativa: Conforme Processo: 01530.000169/2013-67 Declaração de Inexigibilidade em 06/02/2013 . ANA AMELIA DE CARVALHO VELLOSO . Coordenadora do Gabinete da Presidência . Ratificação em 06/02/2013 . ANTONIO CARLOS GRASSI . Presidente da Funarte . Valor Global: R$ 10.000,00 . CNPJ CONTRATADA : 05.778.530/0001-89 GUTORUOCC O PRODUCOES CULTURAIS LTDA - ME.

(SIDEC - 07/02/2013) 403201-40402-2012NE800025

EXTRATO DE INEXIGIBILIDADE DE LICITAÇÃO Nº 1016/2013 - UASG 403201

Nº Processo: 01530000170201391 . Objeto: Contratação da cantora Luciana Rodrigues Melo de Oliveira Levy, em artes Luciana Mello, para realizar uma apresentação,no Espaço Brasil, no âmbito do evento Ano do Brasil em Portugal, na cidade de Lisboa/Portugal, no dia 16 de fevereiro de 2013. Total de Itens Licitados: 00001 . Fundamento Legal: Art. 25º, Inciso III da Lei nº 8.666 de 21/06/1993. . Justificativa: Conforme Processo: 01530.000172/2013-91 Declaração de Inexigibilidade em 06/02/2013 . ANA AMELIA DE CARVALHO VELLOSO . Coordenadora do Gabinete da Presidência . Ratificação em 06/02/2013 . ANTONIO CARLOS GRASSI . Presidente da Funarte . Valor Global: R$ 10.000,00 . CNPJ CONTRATADA : 08.741.133/0001-20 RCS PRODU COES ARTISTICAS E CINEMATOGRAFICAS LTDA - ME.

(SIDEC - 07/02/2013) 403201-40402-2012NE800025

EXTRATO DE TERMO ADITIVO Nº 5/2013 - UASG 403201

Número do Contrato: 41/2011.
Nº Processo: 01530001035/11-00.
PREGÃO SISPP Nº 41/2011 Contratante: FUNDACAO NACIONAL DE ARTES FUNARTECNPJ Contratado: 00729160000176. Contratado : SPOT REPRESENTACOES E SERVICOS -LTDA. Objeto: As partes resolvem alterar o prazo de vigência estabelecido no item 7.1 da cláusula sétima do contrato ora aditado, passando a sua vigência a se encerrar em 28 de fevereiro de 2013. Fundamento Legal: Lei nº8.666/93 . Vigência: 01/02/2013 a 28/02/2013. Data de Assinatura: 31/01/2013.

(SICON - 07/02/2013) 403201-40402-2012NE800025

INSTITUTO BRASILEIRO DE MUSEUS

EXTRATO DE CONVÊNIO

Espécie: Convênio Nº 778691/2012. Convenentes: Concedente : INSTITUTO BRASILEIRO DE MUSEUS, Unidade Gestora: 423002, Gestão: 42207. Convenente : SECRETARIA DE ESTADO DA CULTURA, CNPJ nº 94.235.330/0001-00. Elaborar a documentação básica para possibilitar a posterior obra de restauração e ampliação do Museu Julio de Castilhos. Essa documentação consiste no levantamento arquitetônico, análises de reboco, pintura e fundação, diagnóstico do estado físico, projeto executivo de restauração e ampliação do museu, memorial descritivo, planilha orçamentária, levantamento fotográfico e cronograma-físico financeiro. Além disto, a instalação de uma rede elétrica provisória, dando conta do isolamento para garantia dos demais seValor Total: R$ 325.837,31, Valor de Contrapartida: R$ 70.000,00, Crédito Orçamentário: PTRES: 47082, Fonte Recurso: 0118033902, ND: 33304, Num Empenho: 2012NE800392. Vigência: 20/12/2012 a 02/08/2013. Data de Assinatura: 20/12/2012. Signatários: Concedente : JOSE DO NASCIMENTO JUNIOR, CPF nº 085.318.568-92, Convenente : LUIZ ANTONIO DE ASSIS BRASIL E SILVA, CPF nº 006.456.560-20.

(SICONV(PORTAL) - 07/02/2013)

MUSEU DA REPÚBLICA

AVISO DE LICITAÇÃO PREGÃO ELETRÔNICO Nº 1/2013 - UASG 343018

Nº Processo: 01437000825201217 . Objeto: Pregão Eletrônico - Contratação de pessoa jurídica especializada, para prestação de serviços contínuos de suporte de apoio administrativo para atender às necessidades do Museu da República, de acordo com o Termo de Referência - Anexo I. Total de Itens Licitados: 00001 . Edital: 08/02/2013 de 11h00 às 13h00 e de 14h às 17h00 . Endereço: Rua do Catete, 153 - Catete Catete - RIO DE JANEIRO - RJ . Entrega das Propostas: a partir de 08/02/2013 às 11h00 no site www.comprasnet.gov.br. . Abertura das Propostas: 22/02/2013 às 11h00 site www.comprasnet.gov.br. . Informações Gerais: Maiores informações, contactar o Sr Rogério Maurílio Alecrim Rezende no telefone (21)3235-5113.

ROGERIO MAURILIO ALECRIM REZENDE
Presidente da Comissão Permanente de Licitação

(SIDEC - 07/02/2013) 423002-42207-2013NE800010

INSTITUTO DO PATRIMÔNIO HISTÓRICO E ARTÍSTICO NACIONAL

AVISO DE AUTORIZAÇÃO

O Instituto do Patrimônio Histórico e Artístico Nacional - IPHAN, no uso das competências conferidas pela Deliberação CGEN/MMA nº 279, de 20 de setembro de 2011, publicada no DOU de 9 de novembro de 2011, de acordo com a Medida Provisória nº 2.186 -16, de 23 de agosto de 2001, o Decreto nº 3.945, de 28 de setembro de 2001, e demais normas atinentes, concedeu AUTORIZAÇÃO de Acesso a Conhecimento Tradicional Associado ao Patrimônio Genético, para fins de pesquisa científica, à Universidade Federal de Juiz de Fora - UFJF, em conformidade com o Processo nº 01450.010839/2012-62:

Projeto - Estudo Etnobotânico em Comunidades Quilombolas da Zona da Mata Mineira.

Objetivo geral da pesquisa - O estudo fundamenta-se em: registrar, documentar e resgatar os conhecimentos tradicionais relativos às plantas pelas comunidades quilombolas mineiras de São Pedro de Cima, Colônia do Paiol, São Sebastião da Boa Vista e São Bento, avaliando a erosão cultural nestas comunidades. Identificar a diversidade de vegetais utilizados, bem como o manejo dos mesmos estabelecendo comparações etnográficas, culturais e socioambientais das referidas comunidades.

Comunidade envolvida - Comunidades Quilombolas de São Pedro de Cima, Colônia do Paiol, São Sebastião da Boa Vista e São Bento

Localização - Municípios de Divino, Bias Fortes, Santos Dumont, Minas Geras-MG.

Validade da autorização - março/2014

JUREMA MACHADO
Presidenta do Instituto

SUPERINTENDÊNCIA NO AMAPÁ

EXTRATO DE CONTRATO Nº 7/2012 - UASG 343041

Nº Processo: 01424000031201294.
TOMADA DE PREÇOS Nº 1/2012 Contratante: INSTITUTO DO PATRIMONIO HISTORICO-E ARTISTICO NACIONAL. CNPJ Contratado: 06992587000149. Contratado : ESTILO NACIONAL LTDA - EPP -Objeto: Serviços especializados para elaboraçãodo inventário nacional das referências culturais do Marabaixo do Amapá, de modo a subsidiar a formulação e execução de Plano de Salvaguarda ea elaboração de um dossiê para encaminhamento de proposta de registro do referido bem, como Patrimônio Cultural do Brasil. Fundamento Legal: Tomada de Preço com vulcro noArt. 22, II da Lei 8.666/93. Vigência: 21/12/2012 a 07/12/2013. Valor Total: R$198.178,56. Fonte: 100000000 - 2012NE800085. Data de Assinatura: 21/12/2012.

(SICON - 07/02/2013) 343026-40401-2012NE800077

EXTRATO DE TERMO ADITIVO Nº 1/2013 - UASG 343041

Número do Contrato: 1/2012.
Nº Processo: 01424000106201156.
PREGÃO SISPP Nº 1/2011 Contratante: INSTITUTO DO PATRIMONIO HISTORICO-E ARTISTICO NACIONAL. CNPJ Contratado: 03100313000190. Contratado : MARIA RODRIGUES DA SILVA - EPP -Objeto: Prorrogação da vigência do contrato pormais 12 (doze) meses consecutivos, e a repactuação dos valores iniciais com base na Covenção Coletiva de trabalho 2012/2012, com registro noMTE: AP000084/2012, conforme previsto nas clausulas do contrato. Fundamento Legal: Art. 65 da Lei 8.666/93 . Vigência: 02/01/2013 a 02/01/2014. Valor Total: R$18.327,60. Fonte: 100000000 - 2012NE800005. Data de Assinatura: 02/01/2013.

(SICON - 07/02/2013) 343026-40401-2012NE800077

EXTRATO DE TERMO ADITIVO Nº 2/2013 - UASG 343041

Número do Contrato: 2/2012.
Nº Processo: 01424000106201156.
PREGÃO SISPP Nº 1/2011 Contratante: INSTITUTO DO PATRIMONIO HISTORICO-E ARTISTICO NACIONAL. CNPJ Contratado: 05393490000157. Contratado : LUNIC LTDA - EPP -Objeto: Prorrogação da vigência do contrato pormais 12 (doze) meses consecutivos, e a repactuação dos valores iniciais com base na Covenção Coletiva de trabalho 2012/2012, com registro noMTE: AP000084/2012, conforme previsto nas clausulas nona e décima primeira do contrato. Fundamento Legal: Art. 65 da Lei 8.666/93 . Vigência: 02/01/2013 a 02/01/2014. Valor Total: R$39.016,32. Fonte: 100000000 - 2012NE800011. Data de Assinatura: 02/01/2013.

(SICON - 07/02/2013) 343026-40401-2012NE800077

EXTRATO DE TERMO ADITIVO Nº 2/2012 - UASG 343041

Número do Contrato: 4/2012.
Nº Processo: 01424000106201156.
PREGÃO SISPP Nº 1/2011 Contratante: INSTITUTO DO PATRIMONIO HISTORICO-E ARTISTICO NACIONAL. CNPJ Contratado: 03100313000190. Contratado : MARIA RODRIGUES DA SILVA - EPP -Objeto: Prorrogação da vigência do contrato pormais 12 (doze) meses consecutivos, e a repactuação dos valores iniciais com base na Covenção Coletiva de trabalho 2012/2012, com registro noMTE: AP000084/2012, conforme previsto nas clausulas nona e décima primeira do contrato. Fundamento Legal: Art. 65 da lei 8.666/93 . Vigência: 02/01/2013 a 02/01/2014. Valor Total: R$28.260,60. Fonte: 100000000 - 2012NE800004. Data de Assinatura: 31/12/2012.

(SICON - 07/02/2013) 343026-40401-2012NE800077

EXTRATO DE TERMO ADITIVO Nº 3/2013 - UASG 343041

Número do Contrato: 5/2012.
Nº Processo: 01424000110201114.
TOMADA DE PREÇOS Nº 2/2011 Contratante: INSTITUTO DO PATRIMONIO HISTORICO-E ARTISTICO NACIONAL. CNPJ Contratado: 34927285000122. Contratado : INSTITUTO DE PESQUISAS CIENTIFICASE TECNOLOGICAS DO ES. Objeto: Prorrogação da vigência do contrato pormais 90 (noventa) dias. Fundamento Legal: Art. 57 §1, II da Lei 8.666/93. Vigência: 07/01/2013 a 08/04/2013. Data de Assinatura: 07/01/2013.

(SICON - 07/02/2013) 343026-40401-2012NE800077

SUPERINTENDÊNCIA EM SANTA CATARINA

EXTRATO DE CONTRATO Nº 1/2013 - UASG 343011

Nº Processo: 01510002312201211.
PREGÃO SISPP Nº 5/2012 Contratante: INSTITUTO DO PATRIMONIO HISTORICO-E ARTISTICO NACIONAL. CNPJ Contratado: 03814774000144. Contratado : CANADENSE - ADMINISTRACAO E - SERVICOS LTDA - EPP. Objeto: Prestação de serviços continuados de limpeza e cnservação com fornecimento de materiais de limpeza e higiene e equipamentos, a serem executados no Prédio da Antiga Alfandega, em Florianópolis/SC. Fundamento Legal: Lei 10.520/2002, Decreto 3.555/2000, Decreto 5.450/2005 e lei 8.666/93. Vigência: 04/02/2013 a 04/02/2014. Valor Total: R$28.998,00. Fonte: 100000000 - 2013NE800001. Data de Assinatura: 04/02/2013.

(SICON - 07/02/2013) 343026-40401-2013NE800077
